# Former Incarceration, Time Served, and Perceived Oral Health among African American Women and Men

**DOI:** 10.3390/ijerph191912906

**Published:** 2022-10-08

**Authors:** Ryan D. Talbert, Emma D. Macy

**Affiliations:** 1 Department of Sociology, University of Connecticut, Storrs, CT 06269, USA; 2 Department of Human Development and Family Sciences, University of Connecticut, Storrs, CT 06269, USA

**Keywords:** incarceration, prison term, oral health, African Americans, gender, teeth, gums

## Abstract

A large body of research has documented the far-reaching health consequences of mass incarceration in the United States. Yet, less scholarship has examined the relationship between former incarceration and oral health, a key reflection of health and disease occurring within the rest of the body. Using data extracted from the National Survey of American Life (n = 3343), this study examines associations among former incarceration status, duration of detention, and self-reported oral health among African American women and men. Results from gender-stratified ordered logistic models reveal that formerly incarcerated African American men and women experience significantly poorer oral health than their never incarcerated counterparts even after controlling for important social determinants of health. Furthermore, oral health is curvilinearly associated with the length of time that men are incarcerated such that odds of poor health decrease as detention duration increases up to approximately 15 years incarcerated. After 15 years of detainment, the odds of poor health tend to increase as duration increases. Findings extend research identifying gendered spillover health consequences of contact with the criminal legal system. Health professionals and policymakers should be conscious of incarceration as an important deleterious experience for the immediate and long-term condition of people’s teeth, mouth, and gums.

Despite decarceration efforts in recent decades, the United States remains a world leader in incarceration with an imprisonment rate of 358 per 100,000 residents [1,2]. Research has demonstrated that people ensnared in the sprawling US criminal legal system experience extensive social, psychological, and economic consequences [3]. Mass incarceration reflects the disproportionate incarceration of specific social groups and comparably high incarceration rates historically and internationally [4]. In 2020, Black men were incarcerated at a rate 5.7 times white men, and Black women were 1.7 times more likely than white women to experience incarceration [1]. A large and growing body of literature documents the impacts of mass incarceration on US health disparities. Studies show that mass incarceration is a contributor to higher mortality, worse mental health patterns, and increased rates of communicable and chronic disease [5,6,7,8,9,10,11,12,13]. Despite the breadth of the literature, few studies examined whether incarceration is associated with oral health, which encompasses the condition of people’s teeth, mouth, and gums. This omission is notable considering that oral health is an integral part of one’s overall health status and operates as a mirror of health and disease occurring within the rest of the body [14,15,16]. This study extends the literature by examining the association among former incarceration, duration of detention, and oral health among African American women and men. 

Incarceration reflects confinement in a penal facility including federal or state prisons, or county or city jails. Experiencing incarceration is likely to deteriorate oral health for several reasons. First, despite incarcerated Americans being guaranteed the right to healthcare according to the Eighth Amendment of the US Constitution, penal healthcare systems more often provide reactive medical care (e.g., responding to health emergencies) rather than preventative health services (e.g., health screening exams) [8,17,18,19]. Hence, the subpar quality of healthcare services for those incarcerated may lead to poorer oral health while incarcerated and after release. Second, threats to safety, meals with insufficient nutritious value, and lack of quality oral hygiene products while incarcerated likely contribute to worsening oral health, and these patterns may worsen the longer a person is confined [20,21]. Third, following release, formerly incarcerated adults face challenges in accessing and receiving oral healthcare services due to a lack of transportation, health insurance, or sufficient medication to manage health conditions that have oral health ramifications [16,22,23]. Hence, comparing adults with and without former incarceration status is important for understanding oral health disparities given that reentry into society necessitates navigating the US healthcare system (e.g., obtaining insurance, locating providers, and scheduling appointments).

The present study offers three contributions to the research literature. First, this study focuses on variation in self-reported oral health status among African American adults. While a preponderance of studies found that incarceration is associated with poorer health, [5,6,7,8] fewer studies examined whether this pattern holds for oral health, a key marker of overall health status [14,15,16]. Furthermore, Black Americans typically experience poorer oral health than people of other ethnoracial backgrounds [24]. However, we know less of whether disproportionate exposure to incarceration for Black Americans is a contributing factor to persistently poorer oral health patterns. Identifying the significance of incarceration for oral health is important considering that research identifies innumerable immediate and long-term consequences in other domains (e.g., labor market, housing, and education) [25,26]. By extending research documenting the spillover consequences of contact with the legal system, this study aims to inform health professionals of incarceration as a potentially important deleterious experience for oral health.

Second, this study examines two indicators of former incarceration status including whether one has ever been detained in a prison or jail, as well as the duration of one’s detention. Studies examining associations between incarceration and health typically focus on single indicators of incarceration status (i.e., yes or no) to the neglect of additional measures that may add supplementary information about one’s experience (e.g., years served) [5,6]. Research shows that any incarceration experience is associated with worse health consequences than the length that one is incarcerated [7,27]. However, the literature has sparsely tested whether these findings hold for oral health. Therefore, examining incarceration experience and detention duration offers an important contribution to the literature. Third, this study identifies associations between incarceration and oral health among men and women separately. To this end, some studies found that women experience worse health consequences from incarceration than men, and we interrogate this possibility for oral health. 

## 1. Background and Theory

### 1.1. Mass Incarceration and Health Disparities

Two percent of all Black men in the United States are incarcerated [1]. Because the criminal legal system in the US is so vast, it shapes innumerable social domains including health. Research documents the profoundly negative effects of mass incarceration for health disparities [5,6,7,8,9,10,13,28,29,30]. Moreover, after release, incarceration affects the long-term health of those once ensnared in the system due to greater financial strain, the difficulties of reentering society, and the stigma attached to a criminal record [5,6]. Despite the voluminous literature documenting the ill-health effects of incarceration, we know less about the association between incarceration and oral health. Among the few studies closely related to the topic, findings show that incarcerated adults face greater struggles accessing oral care [20]. In other words, there is reason to anticipate that former incarceration is associated with poorer oral health compared to similarly positioned people without incarceration experience. 

### 1.2. Incarcerated Populations and Oral Health

The oral health literature typically does not incorporate incarcerated populations, which has led to a relatively small boy of literature on incarceration and oral health. Of the studies inclusive of the carceral world, incarcerated people generally experience poorer oral health than nonincarcerated people [31,32]. One contributing factor is that those who are imprisoned struggle accessing quality healthcare, which includes oral healthcare [18,20,33]. Research shows there is a general lack of dental care and support in penal facilities for currently incarcerated people [31,34,35,36,37]. Unclear US federal and state guidelines on dental policy and procedures for prisons lead to a lack of services for incarcerated people [13]. As a consequence, the prevalence of treatable and preventable diseases such as periodontitis is high among those who are incarcerated and continues to be a challenge for those who need care [38,39]. Furthermore, among inmates, at least one study has found that longer detention durations are associated with progressively decaying dental health [34]. This finding is notable considering studies typically identify that experiencing incarceration of any amount of time is worse for health than the length of incarceration [7,27]. Ultimately, data limitations make it difficult to assess oral health for people who have experienced incarceration in the United States, which highlights the importance of the present study’s foci. 

*Oral Health among Incarcerated Black Men*. Incarcerated Black Americans face additional structural barriers to maintaining oral health and accessing treatment beyond the barrier that incarceration generates [34,40]. In other words, racial disparities in oral health occur not only in the general public to the disadvantage of Black populations, but also within the prison system. A consistent finding in the few studies that have documented racial disparities in oral health is that Black inmates are more likely than their white counterparts to have decaying teeth [34,41]. The present study builds on these insights to identify whether former incarceration status factors into variation in oral health among African American men and women specifically.

*Oral Health among Incarcerated Black Women*. Among incarcerated populations, evidence shows that women’s healthcare needs including oral healthcare are typically more underserved than men [42]. In addition, incarcerated women from racially marginalized backgrounds more often experience oral pain than their white counterparts [40]. Said differently, incarcerated women are generally less likely to have their healthcare needs met, and this gender disadvantage is worse for women of color [43]. Poorer access to treatment and worse quality of care on average leads incarcerated women to hold more negative views of the treatment they receive while in prison [33,42]. Lack of proper oral healthcare has immediate and long-term ramifications. One study conducted outside of the US found that incarcerated women’s perception of their oral health was strongly associated with their quality of life including perceived physical discomfort, psychological disability, and social disadvantage [40]. Thus, research demonstrates the struggles that many incarcerated women face with receiving proper dental healthcare, and studies document the importance of care for maintaining oral health for a person’s quality of life. 

### 1.3. Predictors of Post-Release Oral Health

Maintaining oral health and hygiene encompasses routine dental visits, transportation access to care, and having health insurance plans that cover dental health [22,44]. Moreover, despite the importance of wellness checks, disparities in access to care exist with considerable health consequences. Unfortunately, dental care creates one of the highest financial barriers in comparison to other healthcare services, and insurance plans sometimes do not cover dental health [45]. For formerly incarcerated people, the lack of publicly funded programs to assist with covering the cost of dental care creates challenges in accessing and financing care [45]. Routine dental visits often involve 6 month wellness checks, which studies show are clinically beneficial for people [46]. According to a meta-analysis, less frequent dental visits are associated with an increase in dental decay and tooth loss, as well as a lower perceived quality of life [46]. Regular oral wellness checks are important for identifying, treating, and managing some chronic diseases. For instance, evidence suggests that the tumor size and growth of many oral cancers increase with a decrease in dental visits [46]. Regardless of incarceration status, white Americans are on average more likely to receive preventative dental care, while less than half of Black Americans visit a dentist at least annually [47]. Consequently, inequitable access to care for Black Americans is compounded in the event that a person is formerly incarcerated.

Once a person is no longer incarcerated, reintegration into society remains challenging given the stigma associated with imprisonment and a criminal record [40]. Even if a person successfully navigates the healthcare system to schedule an appointment and visit a healthcare provider, almost half of people who have been incarcerated report experiencing discrimination from healthcare workers [48]. Thus, incarceration can cause long-term negative impacts on oral health via the stigma and discrimination one experiences in the healthcare system. Ultimately, formerly incarcerated adults are expected to have poorer oral health than their never incarcerated counterparts.

## 2. Summary and Hypotheses

The present study examines the association between incarceration experience and self-reported oral health among African American men and women. On the basis of the background research and tenets of our conceptual framework, formerly incarcerated people face extended challenges in accessing quality dental healthcare. Hence, *Hypotheses 1a,b* anticipate that (*a*) women and (*b*) men with a history of incarceration will have worse oral health than their never incarcerated counterparts. While foregoing studies typically show that any incarceration experience is worse for health than the amount of time one is incarcerated, some studies identified that oral health worsens as detention duration increases. Thus, *Hypotheses 2a,b* anticipate that longer durations of incarceration will be associated with poorer oral health among (*a*) women and (*b*) men. 

### 2.1. Data and Methods 

#### 2.1.1. Data

Survey data for this study were extracted from the National Survey of American Life (NSAL), a nationally representative household probability survey collected between 2001 and 2003. The NSAL included 3570 non-Hispanic Blacks, 1438 non-Hispanic Black Caribbeans, 891 non-Hispanic Whites, and 183 Hispanics. The survey emphasized the nature of race and ethnicity within the US Black population by interviewing national samples of African American (n = 3570) and Caribbean (n = 1623) immigrant and older generation populations [49,50]. Full details about the sample and survey were published elsewhere [49,50]. Most interviews were conducted face-to-face in English. The NSAL data are well positioned to address the present study’s aims for at least three reasons. First, the NSAL is one of few surveys to include questions on former incarceration, detention duration, and perceived oral health at the height of mass incarceration in the US. Second, the NSAL includes a nationally representative sample of African Americans. The representative and adequate sample allows for meaningfully examining variation in oral health among African American men and women separately [51,52,53]. Third, the data represent one of the most comprehensive social surveys of US-residing people of African descent ever conducted [50,54]. Thus, the data align well with the present foci. 

#### 2.1.2. Dependent Variable

*Perceived Oral Health Status.* The dependent variable, self-rated oral health, is a commonly used subjective global assessment of oral health status. We use this measure for three reasons. First, the measure aligns with tooth loss, oral pain and discomfort, and dentists’ professional ratings of oral health [55,56,57]. Thus, the measure provides a valid, reliable, and cost-efficient way to assess oral health [58,59,60]. Second, the measure is a strong predictor of other health measures including hypertension, diabetes, and obesity [56,61]. Third, the item we use is commonly used in the research literature and has been validated for study population [62,63,64]. Self-rated oral health derives from answers to the following question: “How would you rate the overall condition of your teeth, gums, and mouth at the present time?” Answers were coded into five categories such that poor = 1, fair = 2, good = 3, very good = 4, and excellent = 5. 

#### 2.1.3. Independent Variables

*Formerly Incarcerated*. Formerly incarcerated identifies whether a person has ever spent time in a jail or prison (yes = 1). 

*Years Spent Incarcerated*. Incarceration duration reflects the length of time in total years that a person experienced incarcerated in the past. We utilized a squared term in multivariable models given that the measure is overdispersed for women and men. 

#### 2.1.4. Covariates

The present study incorporates several measures important to research on criminal legal contact and health including age, education, employment, marital status, and health insurance status and type [28,29,65,66]. Age measures years since birth. Education measures completed years of schooling. Employment measures whether people are currently working (yes = 1). Health insurance coverage includes whether a person has no insurance (yes = 1), federally funded insurance (yes = 1), or employee sponsored insurance (yes = 1). No insurance served as the reference group in multivariable models. We created three marital status categories: married/cohabiting, formerly married (i.e., divorced, widowed, or separated), and never married. Married/cohabiting served as the reference group in multivariable models. 

#### 2.1.5. Methods

We began our analytic strategy by estimating descriptive statistics for African American adults for all study variables. Because we were interested in heterogeneity among the two groups, we stratified all analyses by gender. This study utilized ordered logistic regression models to examine self-rated oral health given its Likert scale construction [67,68]. We estimated four total models. The first models for women and men estimate the association between former incarceration status and oral health while controlling for covariates (i.e., Models 1 and 3). The second models for each gender group estimate the association between duration of incarceration and self-reported oral health among people who have any history of incarceration (i.e., Models 2 and 4). Results from regression models are presented using exponentiated coefficients for ease of interpretation (i.e., odds ratios). All statistics presented are corrected for the complex sampling design of the National Survey of American Life. Reference groups for multicategory measures are indicated using the abbreviation ref. Bayesian Information Criterion (BIC) statistics are calculated for each model.

## 3. Results

### 3.1. Descriptive Statistics

Descriptive statistics are presented in Table 1. On average, women rated their oral health as poorer than men (women mean = 3.08, sd = 1.21; men mean = 3.18, sd = 0.98; *p* < 0.05). Age ranged from 18 to 93 (women *mean* = 42.64, *sd* = 17.52; men *mean* = 41.58, *sd* = 14.12; *p* < 0.05). Women averaged 12.48 (*sd* = 2.59) years of education, and men averaged 12.50 (*sd* = 2.21). Approximately 73% of men were employed, which was significantly higher than the 64% of women that were employed (*p* < 0.05). The percentage of women who had federal program insurance (26%) was significantly higher than the percentage of men who did (15%; *p* < 0.05). The percentage of men who had employee-based insurance (62%) was significantly higher than the percentage of women who did (53%; *p* < 0.05). African American men were more likely to fall into the married/cohabiting category (50%; *p* < 0.05), while women were more likely to occupy the formerly married group (32%; *p* < 0.05).

### 3.2. Incarceration, Duration, and Dental Health

Results from ordered logistic regression models estimating oral health for African American women and men in the National Survey of American Life are presented in Table 2. Model 1 estimates self-rated oral health based on former incarceration status and covariates. Results show that formerly incarcerated women on average report poorer oral health by a factor of 0.65 (*se* = 0.10; *p* < 0.01). Older women also reported poorer oral health on average than younger women (*OR* = 0.98, *se* = 0.01; *p* < 0.001). Additionally, a year increase in education was associated with an improvement in oral health by a factor of 1.09 (*se* = 0.02; *p* < 0.001). On average, women with employment-based health insurance typically had better oral health than those with no insurance (*OR* = 1.32, *se* = 0.14; *p* < 0.05), and women who were formerly married had worse health than married/cohabiting women (*OR* = 0.81, *se* = 0.08; *p* < 0.05). 

**Figure 1:**
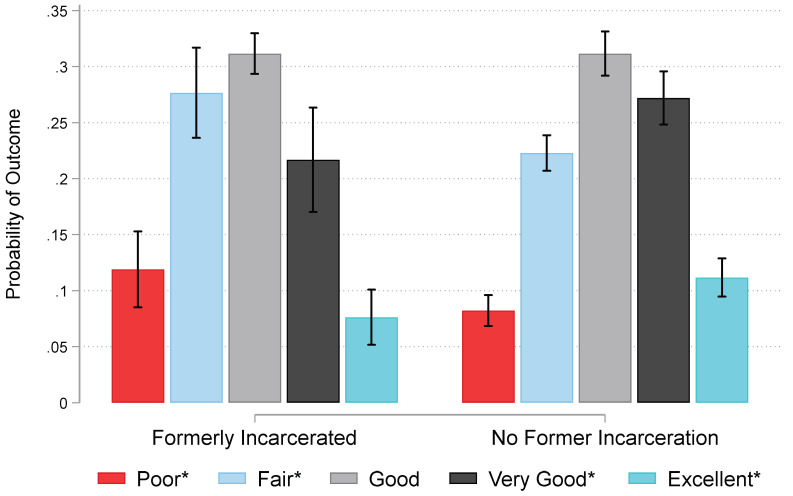
Predictions of Oral Health among African American Women in the National Survey of American Life, 2003. *Note*: Fully-adjusted estimates generated from Model 1 in Table 2 (n = 2144). Probabilities of poor and fair health are significantly higher for formerly incarcerated women. Probabilities of very good and excellent health are significantly lower for formerly incarcerated women. * Probabilities of an outcome are significantly different for formerly and never incarcerated women at *p* < 0.05.

To present the association visually, Figure 1 graphs fully adjusted predictions of self-reported oral health for African American women across former incarceration status. Figure 1 shows that formerly incarcerated women are significantly more likely to rate their oral health as poor (12% probability versus 8%; *p* < 0.05) or fair (28% probability versus 22%; *p* < 0.05), and significantly less likely to report their oral health as very good (22% probability versus 27%; *p* < 0.05) or excellent (8% probability versus 11%; *p* < 0.05). In other words, former incarceration is associated with worse perceived oral health among women. In addition, Model 2 presented in Table 2 focuses on African American women with a history of incarceration to identify the association between duration of detention and oral health. Results show no significant associations among years spent incarcerated, the squared term, and self-reported oral health.

**Figure 2:**
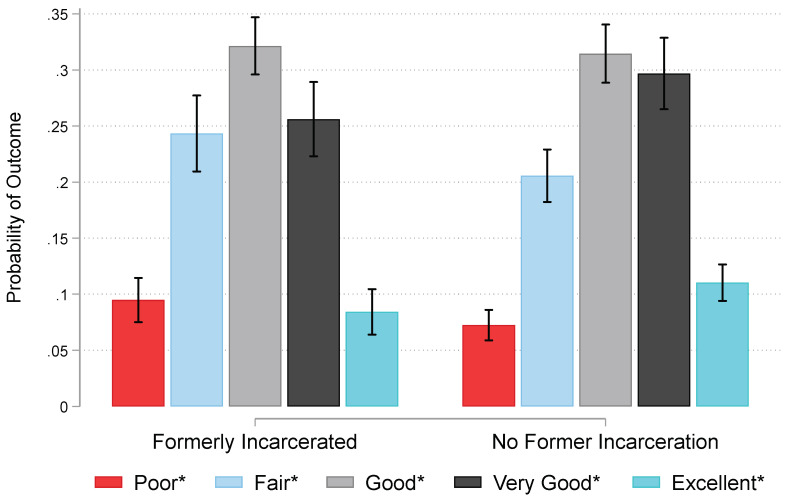
Predictions of Oral Health among African American Men in the National Survey of American Life, 2003. *Note*: Fully-adjusted estimates generated from Model 3 in Table 2 (n = 1166). Probabilities of poor, fair, and good health are significantly higher for formerly incarcerated men. Probabilities of very good and excellent health are significantly lower for formerly incarcerated men. * Probabilities of an outcome are significantly different for formerly and never incarcerated men at *p* < 0.05.

Model 3 shows results for African American men (Table 2). Formerly incarcerated men on average rate their oral health worse by a factor of 0.74 (*se* = 0.09; *p* < 0.05). Older men (*OR* = 0.98, *se* = 0.01; *p* < 0.001) and less educated men (*OR* = 1.09, *se* = 0.04; *p* < 0.01) typically experience poorer oral health than younger and more educated men. Moreover, employed men have better oral health than their unemployed counterparts on average (*OR* = 1.53, *se* = 0.25; *p* < 0.05). Figure 2 graphs fully adjusted predictions of oral health for African American men by former incarceration status. On average, formerly incarcerated men are more likely to report their oral health as poor, fair, or good than their never incarcerated counterparts, and less likely to report their oral health as very good or excellent (all at *p* < 0.05). Formerly incarcerated men are 9% likely to have poor oral health, 24% likely to have fair health, and 32% likely to have good health. Contrarily, never incarcerated men have a 30% probability of very good oral health and an 11% probability of excellent health. 

**Figure 3:**
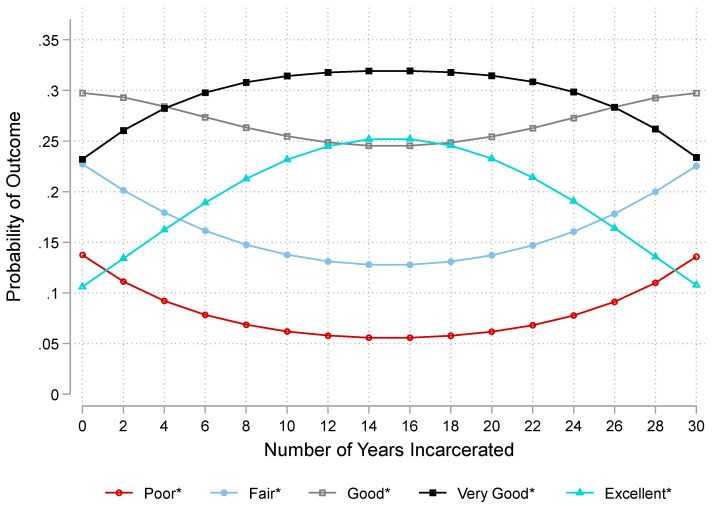
Predictions of Oral Health by Number of Years Incarcerated among Formerly Incarcerated African American Men in the National Survey of American Life, 2003. *Note*: Fully-adjusted estimates generated from Model 4 in Table 2 (n = 252). * Probabilities of an outcome across years incarcerated are significantly different for zero at *p* < 0.05 indicating curvilinear associations.

Model 4 in Table 2 incorporates years incarcerated and its squared term for African American men with a history of incarceration. Results show that an increase of 1 year incarcerated is associated with higher odds of reporting better oral health by a factor of 1.16 (*se* = 0.06; *p* < 0.05). However, the squared term indicates that the odds change directions such that oral health tends to deteriorate for those with the longest durations (*OR* = 0.99, *se* = 0.00; *p* < 0.01). To make sense of these patterns, Figure 3 shows the adjusted predictions for self-reported oral health for formerly incarcerated African American men across years of incarceration. Two notable patterns emerge. First, the slopes estimating probabilities of each of the five reported health options across years of incarceration are all statistically different from zero, which statistically verifies that each of the lines is curvilinear. Said differently, the association between years incarcerated and oral health changes across years of incarceration. Second, the probabilities of reporting poor, fair, or good health tend to decrease across years incarcerated until approximately 15 years at which point the graph indicates that probabilities increase for these outcomes. By contrast, probabilities of very good or excellent health increase across time up until about 15 years at which point the oral health tends to worsen.

## 4. Discussion

This study examined the association between incarceration and oral health. On the basis of background research and tenets of our conceptual framework, we first expected that formerly incarcerated African American (*a*) women and (*b*) men would have poorer oral health than their never incarcerated counterparts (*Hypotheses 1a,b*). Results shown in Table 2 and Figure 1 and Figure 2 support Hypotheses 1a,b such that formerly incarcerated women and men on average rate their oral health as poorer than their never incarcerated counterparts. Moreover, we anticipated that longer timespans of incarceration would be associated with poorer oral health for (*a*) women and (*b*) men (*Hypotheses 2a,b*). Results in Table 2 and Figure 3 did not offer support for Hypotheses 2a,b. We found evidence for a curvilinear association between detention duration and oral health such that odds of having poor oral health decrease across time up to approximately 15 years incarcerated after which time each additional year a person spends incarcerated is associated with progressively poorer oral health (Figure 3). 

We found that incarceration is associated with worse oral health for African American men and women. The literature offers explanations for these patterns. Following release, formerly incarcerated adults may face challenges in accessing and receiving oral healthcare services due to a lack of transportation, health insurance, or sufficient medication to manage health conditions [16,22,23]. In fact, transportation remains one of the main barriers to acquiring proper dental care for older adults [44]. Lack of transportation impedes regular dental visits, which are vital to proper dental health [46]. Moreover, formerly incarcerated people may lack dental insurance, and dental care creates one of the highest financial barriers in comparison to other healthcare services [45]. The lack of publicly funded programs offers people little recourse to access oral healthcare in the absence of dental insurance and expendable income. Furthermore, in cases where people successfully obtain insurance that covers dental healthcare, one will still need to navigate the system to locate a provider and schedule an appointment. Additionally, formerly incarcerated people are also typically released without sufficient medication to manage health conditions [16,22]. Not having medications to manage chronic health conditions can worsen oral health. 

The length of incarceration was curvilinearly associated with oral health among African American men such that spending some time incarcerated is associated with better oral health than those that spend very little time incarcerated. Findings from this research add to a line of studies identifying complex results on the association between criminal legal contact and health specifically among Black men. Some studies found that, while incarceration tends to worsen health for most race–gender groups, Black men do not as often experience the health-worsening effects of incarceration or other forms of criminal legal contact (e.g., arrest) [6,10,11,12]. Scholars posit that incarceration may not always significantly harm Black men’s health due to incarceration shielding them from car crashes, lethal violence, and inaccessible healthcare services given that insurance in the US is often tied to one’s employer [6,11,12]. However, we also found there was a turning point such that the greatest number of years incarcerated is associated with poorer oral health. We propose several explanations for these findings. First, however limited, these men have access to some degree of healthcare services. Some substandard healthcare while incarcerated is likely better for oral health than having no healthcare while not incarcerated. Second, prolonged exposure to threats to safety, meals with insufficient nutritious value, and lack of quality oral hygiene products may worsen oral health after a period of successfully navigating these conditions and strains [20,21]. Third, once released, reintegration into society may present more challenges the longer that one is incarcerated [40]. In other words, the stigma attached to former incarceration may be greater for those with the longest detention durations, and the stigma may manifest in substandard and discriminatory treatment by medical professionals [48].

The present study contributes to existing research but is limited in multiple ways. First, it is likely that incarceration exacerbates preexisting health disparities such that those who become incarcerated are often a population already disadvantaged on the basis of race/ethnicity and socioeconomic status [26]. Future research would benefit from testing the effect of incarceration on oral health while accounting for oral health status prior to incarceration. Doing so would allow a fuller test of incarceration’s longitudinal oral health effects. Second, the data used in this study are a unique and well-positioned source of information to address our research questions. However, the datedness of the data means that future work may build on these findings to ascertain incarceration status, ethnoracial identification, and oral health among a more recent sample of African American adults. Future work may also benefit from use of digital technologies to ascertain health status and to ensure healthcare services are successful [69]. Third, we utilized a common measure of perceived oral health status [62,63,64]. Nonetheless, future studies would benefit from examining additional indicators of oral health status including the presence of oral disease, tooth loss, or functional limitations. Including these additional measures may offer a more comprehensive assessment of the oral health impacts of incarceration. 

## 5. Conclusions

Despite decarceration efforts, the US remains a world leader in incarceration. The present study found that former incarceration is associated with poorer perceived oral health among African American women and men. In doing so, this study adds to research identifying the criminal legal system as having spillover consequences for racial health disparities. While oral health is typically isolated from consideration of a person’s overall health status, oral health reflects health and disease occurring within the rest of one’s body. To this end, medical professionals and policymakers should be aware of incarceration as a consequential experience for the condition of people’s teeth, mouth, and gums. Moreover, healthcare services available within prison systems should include regular oral health examinations, screening test for dental caries, and routine cleanings. Oral health remains integral to overall health, and it would benefit formerly incarcerated men and women to have had regular access to oral healthcare while they were incarcerated. Furthermore, to ensure equitable access to good oral health and healthcare, it remains imperative for federal and US state governments to divest from punitive criminal justice policies that disproportionately incarcerate African American men and women for longer durations often in overcrowded and underserved facilities.

## Figures and Tables

**Table 1:** Descriptive statistics for African American women and men in the National Survey of American Life, 2003.

	African American Women		African American Men	
*Variables*	Mean/%	SD	Mean/%	SD
*Oral Health Status*				
Self-rated oral health * (*range* 1–5, 5 = excellent)	3.08	(1.21)	3.18	(0.98)
*Incarceration Experience*				
Formerly incarcerated *	6.33%	—	21.05%	—
Years spent incarcerated * ^a^ (*range* 0–30.34)	1.81	(3.50)	2.35	(4.11)
*Covariates*				
Age (in years; *range* 18–93)	42.64	(17.52)	41.58	(14.12)
Education (in years; *range* 4–17)	12.48	(2.59)	12.50	(2.21)
Employed (yes = 1) *	63.68%	—	72.67%	—
No insurance (yes = 1)	20.02%	—	23.01%	—
Federal program insurance * (yes = 1)	26.20%	—	15.18%	—
Employee sponsored insurance * (yes = 1)	52.78%	—	61.81%	—
Married/cohabiting (yes = 1) *	35.88%	—	50.30%	—
Formerly married * (yes = 1)	32.25 %	—	19.14%	—
Never married (yes = 1)	31.86%	—	30.56%	—
Sample size	2144		1166	

*Note*: Analyses are corrected for the sampling design. Means and percentages (%) are presented with standard deviations in parentheses (*SD*). * Means/proportions different at *p* < 0.05. ^a^ Only among people who have experienced incarceration.

**Table 2:** Ordered logistic regression models predicting oral health for African American women and men in the National Survey of American Life, 2003.

	Self-Rated Oral Health (*Range* 1–5, 5 = *Excellent*)							
	African American Women				African American Men			
	Model 1		Model 2		Model 3		Model 4	
*Variables*	OR	SE	OR	SE	OR	SE	OR	SE
*Incarceration Experience*								
Formerly incarcerated (yes = 1)	0.65 ^**^	(0.10)	—	—	0.74 ^*^	(0.09)	—	—
Years spent Incarcerated	—	—	0.93	(0.09)	—	—	1.16 ^**^	(0.06)
Years spent Incarcerated	—	—	1.00	(0.00)	—	—	0.99 ^*^	(0.00)
*Covariates*								
Age (in years)	0.98 ^***^	(0.01)	0.94 ^*^	(0.02)	0.98 ^***^	(0.01)	0.98	(0.01)
Education (in years)	1.09 ^***^	(0.02)	1.12 ^*^	(0.02)	1.09 ^**^	(0.04)	1.21 ^*^	(0.10)
Employed (yes = 1)	1.01	(0.14)	0.93	(0.57)	1.53 ^*^	(0.25)	2.28 ^*^	(0.85)
Federal program Insurance (ref = no insurance)	0.79	(0.10)	0.83	(0.51)	0.97	(0.18)	1.22	(0.45)
Employee sponsored insurance (ref = no insurance)	1.32 ^*^	(0.14)	1.43	(0.74)	1.05	(0.17)	1.28	(0.44)
Formerly married (ref = married/cohabiting)	0.81 ^*^	(0.08)	1.13	(0.52)	0.78	(0.12)	0.52 ^*^	(0.17)
Never married (ref = married/cohabiting)	1.13	(0.12)	1.59	(0.57)	1.13	(0.16)	1.46	(0.42)
Sample size	2144		127		1166		252	
BIC	4396.310		318.592		3333.870		773.058	
McFadden Pseudo R^2^	0.031		0.080		0.031		0.060	

*Note*: Analyses are corrected for the sampling design. Odds ratios (*OR*) are presented with rounded standard errors (*SE*) in parentheses for African American women and men. Reference groups for multicategory measures are indicated using the abbreviation ref. Models 2 and 4 only include adults who have ever been incarcerated. BIC indicates the Bayesian Information Criterion. * p < 0.05; ** *p* < 0.01; *** *p* < 0.001 (two-tailed tests).

## Data Availability

The data utilized in this study have special restrictions and are not publicly available. To obtain these data, researchers must agree to the terms and conditions of a Restricted Data Use Agreement in accordance with existing Inter-university Consortium for Political and Social Research servicing policies (https://www.icpsr.umich.edu/web/ICPSR/studies/20240, accessed on 1 October 2022).
